# A Sociocultural Perspective on English-as-a-Foreign-Language (EFL) Teachers’ Cognitions About Form-Focused Instruction

**DOI:** 10.3389/fpsyg.2021.593172

**Published:** 2021-03-31

**Authors:** Qiang Sun, Lawrence Jun Zhang

**Affiliations:** ^1^School of Foreign Languages, Henan Polytechnic University, Jiaozuo, China; ^2^Faculty of Education and Social Work, University of Auckland, Auckland, New Zealand

**Keywords:** teachers’ cognitions, Chinese EFL teachers, form-focused instruction, Sociocultural Theory, foreign language education

## Abstract

There has been much research into teacher beliefs about teaching and learning as seen in the general teacher education literature. In the field of language teacher education, this line of research has been evolving, with the recent trend being streamlined into “teacher cognition” as a generic or umbrella term. Despite increasing amounts of research output so far, research into foreign language teachers’ cognitions about their own teaching and decision-making is still insufficient, particularly with regard to university-level English-as-a-foreign-language (EFL) teachers in China. Drawing on Vygotsky’s Sociocultural Theory, this qualitative research focused on EFL teachers’ cognitions about form-focused instruction in Chinese university settings. It intended to discover how teachers’ cognitions changed when they were expected to teach in actual classrooms and what factors contributed to these changes. Data collected from four teacher-participants through semi-structured interviews, classroom observations and follow-up stimulated recall interviews showed participants’ support for *focus-on-form* instruction, which means they not only paid attention to the grammatical form of the language but also to the meaning it is intended to convey. However, data also showed that the teacher-participants shifted from *focus-on-form* to *focus-on-formS* instruction in actual teaching, which suggests that they might have realized the challenges of carrying out teaching activities surrounding *focus-on-form* and would like to take an easier approach by only teaching the grammar of the language by *focusing on formS*. Such incongruences are interpreted with reference to a plethora of sociocultural factors including traditional Chinese thinking and institutional expectations. The implications of the findings for stakeholders in universities, including faculty members, students, and curriculum developers in similar contexts, are also discussed.

## Introduction

There has been a renewed interest in the study of language teacher cognition in the past three decades (see also Lee, 2009; Mak, 2011; Borg, 2015; Li, 2017; Gao and Zhang, 2020; Li, 2020). Such research gains its popularity again because of scholars’ interest in teacher cognition in mainstream education research where, there is a general recognition that teachers are active, thinking decision-makers who play a pivotal role in shaping classroom teaching (Shulman, 1986; Zhang and Ben Said, 2014). Understanding language teachers’ cognitions is central to understanding their teaching; and research along this line is burgeoning (e.g., Sun, 2017; Clark-Gareca and Gui, 2019; Gao et al., 2020; Yu et al., 2020).

In the field of second language education, a number of studies have examined language teachers’ generic cognitions about language teaching. Studies into teachers’ cognitions about specific curricular domains such as grammar, reading, oral communication, listening, and writing are also reported in the literature (Zhang and Rahimi, 2014; Borg, 2015; Rahimi and Zhang, 2015; Gao, 2019; Zhao, 2019). More recent studies have also examined teachers’ cognitions about online teaching (Gao and Zhang, 2020). Most of them were conducted from a cognitive perspective (Barcelos, 2003(for syntheses and meta-reviews, see Borg, 2015, 2019; Sun and Zhang, 2019; Li, 2020; see also Bao et al., 2016). Such studies are characterized by viewing teachers’ cognitions as a static, internal representation of experience that is resistant to change, and most often data were collected through questionnaires (Dufva, 2003; Sun and Zhang, 2019). However, it is generally acknowledged that teachers’ cognitions are not only cognitive; they are personal, situated and dynamic (Burns et al., 2015; Kubanyiova and Feryok, 2015). Therefore, as is well argued in Li (2020), there is a need for research into language teachers’ cognitions to shift its theoretical lens from a cognitive to a sociocultural perspective (Burns et al., 2015; Kubanyiova and Feryok, 2015; Sun and Zhang, 2019).

English language education in China has gained its momentum since China adopted the policy of opening to the outside world in the 1980s (Wang and Gao, 2008; Li, 2012). However, Chinese University English teaching has been critiqued for its teacher-centred classrooms and traditional Grammar-Translation teaching methods (Zhang, 2015; Li et al., 2020). In response to this issue, the Chinese Ministry of Education (hereafter MOE), have made massive top-down reforms in advancing English Language Teaching (ELT) at the tertiary level (Zhang and Liu, 2014). A recent reform is that the MOE established the *College English Curriculum Requirements* in 2007 as a policy guide for University English teaching (Ministry of Eduation, 2007). The new policy document requires University English teachers to shift away from solely using traditional teaching methods such as Grammar-Translation to embracing some new methods such as form-focused instruction (meaning is primary but grammar is not neglected either), communicative language teaching (CLT) and its more recent version, task-based language teaching (TBLT), which enable teachers not only to teach students linguistic knowledge but also to develop their communicative competence (Richards, 2005).

Framed in Vygotsky’s Sociocultural Theory, this paper explored Chinese university EFL teachers’ cognitions about form-focused instruction in university classrooms. Teachers’ cognitions were examined through data collected from different research tools including semi-structured interviews, classroom observations, and follow-up stimulated recall interviews with reference to specific teaching contexts. Accordingly, this qualitative study was designed and intended to gain an in-depth understanding of teachers’ cognitions and their changes. Given the relative absence of research into Chinese EFL teachers’ cognitions about and practices in teaching English grammar and vocabulary, the main purpose of this study was to have a comprehensive and holistic grasp of teachers’ cognitions, particularly how their conceptualizations and actual implementation of form-focused instruction in university English teaching matched or mismatched and why such incongruences arose if mismatches emerged as a major finding.

## Literature Review

We present a theoretical framework, Sociocultural Theory, which guided the current study. Then we review empirical studies into language teachers’ cognitions and form-focused instruction in the field of second and foreign language education in order to show a research gap that our study aimed to fill.

### Theoretical Framework

Sociocultural Theory (hereafter SCT) was adopted to guide the current study, where mediation is regarded as a key construct. According to SCT, human cognition is created through engagement in social activities and it is the social relationships and the culturally constructed materials and designs, usually referred to as semiotic artifacts that mediate relationships that create unique human thinking (Lantolf, 2006; Johnson, 2009; Li, 2020). Therefore, cognitive development is an interactive process, mediated by the social, cultural, and historical contexts. Knowledge of the world is mediated by means of being situated in a social environment and from which humans gain the representational systems that ultimately turns into the medium or the mediator of thought. As Wertsch (1995) said, “individuals have access to psychological tools and practices by virtue of being part of a sociocultural milieu in which those tools and practices have been and continue to be culturally transmitted” (p. 141). The major goal of a sociocultural approach to mind is to create an account of human mental processes, which recognizes the essential relationship between these processes and their cultural, historical, and institutional settings (Wertsch, 1991). The rationale of using SCT in this study is that teachers’ cognitions about form-focused instruction are explored not only from teachers’ own mental processes but also in social contexts such as the university and the society. In other words, teachers’ cognitions about form-focused instruction might change due to the mediation of cultural, institutional, and historical contexts they live in. SCT has been adopted to understand either teachers’ cognitions or learners’ beliefs about language teaching and learning in other contexts. For example, Yang and Kim (2011) explored second language (L2) learning beliefs in study-abroad contexts. Mohamed (2014) investigated aspects of epistemic beliefs of pre-service and in-service teachers in the United Arab Emirates, and how these beliefs might be related to factors such as teachers’ gender, location (where they live), and the subject they teach (humanities vs. science). In our study, SCT is adopted to explain the incongruence between Chinese university teachers’ cognitions about form-focused instruction reported in interviews and those practices found in classroom observations.

### Teachers’ Cognitions

Research into language teachers’ cognitions emerged and flourished as the study of language teaching shifted its focus from a process-product approach to a teachers’ thinking paradigm (Clark and Peterson, 1986; Calderhead, 1996; Borg, 2003). The new paradigm integrates teachers’ behavior into their thinking underlying their behavior, focusing on what motivates their behavior or action. The assumption of this paradigm is what teachers do in their classroom teaching reflects what they know and believe, and that teacher knowledge and teacher thinking provide an underlying framework to guide their classroom practices (Richards and Lockhart, 1994). However, researchers used multiple terms to define teachers’ thinking such as *beliefs*, *knowledge*, *teaching principles*, *maxims*, *schema*, *cognitions*, and *personal theories* (Freeman, 2002; Borg, 2003, 2019). Following Borg’s (2015) theorization, we use *teachers’ cognitions* as an umbrella term to refer to “the unobservable cognitive dimension of teaching-what teachers know, believe, and think” (Borg, 2003, p. 81).

### Form-Focused Instruction

Form-focused instruction subsuming two categories, *focus-on-formS* and *focus-on-form*, was initially proposed by Long (1991). *Focus-on-formS* instruction is described as the presentation and practice of discrete and decontextualized linguistic rules and structures without any communicative context (Long, 1991; Long and Robinson, 1998). It encompasses traditional grammar instruction and other structural approaches (Long, 1991; Ellis, 2001). Researchers have generally reached agreement on the definition of *focus-on-formS* but they define *focus-on-form* in varied ways. According to Long (1991), *focus-on-form* is a type of instruction that “overtly draws students’ attention to linguistic elements as they arise incidentally, in lessons where the overriding focus is on meaning or communication” (pp. 45–46). Ellis extends Long’s definition of *focus-on-form*, defining it as either drawing students’ attention to linguistic elements as they arise incidentally or to preselected language items in communicative activities (Ellis, 2001, 2017). This type of *focus-on-form* is similar to *focus-on-formS* in that it still attends to pre-selected forms, but the focus is to communicate.

Spada (1997) defined form-focused instruction as “pedagogical events which occur within meaning-based approaches to L2 instruction but in which a focus on language is provided in either spontaneous or predetermined ways” (p. 73). This definition is similar to Long’s (1991) and Ellis’s (2001) conceptualizations of *focus-on-form*, but excludes *focus-on-formS* (Spada, 2011). Researchers conducted a series of studies to examine the effects of two types of form-focused instruction according to Spada’s definition of form-focused instruction. For example, Spada et al. (2014) compared the effects of two types of form-focused instruction (FFI), isolated and integrated FFI, on second language (L2) learning and their potential contributions to the development of different types of L2 knowledge. Other researchers like Spada et al. (2009) attempted to develop a questionnaire to explore teachers’ preference for two types of form-focused instruction (FFI): isolated and integrated FFI. The results indicated that the questionnaire was both a valid and reliable measure of isolated and integrated FFI. These studies are all conducted around the types of form-focused instruction: isolated and integrated FFI according to Spada’s definition of form-focused instruction. In this study, we adopted Ellis’ conceptualization of form-focused instruction, classifying *focus-on-form* and *focus-on-formS* according to teachers’ classroom instruction, either grammar instruction or vocabulary teaching, occurring in meaning-based activities or not (Shegar et al., 2013; see also Zhang and Zhang, 2021). If teachers arrange meaning-based activities with a primary purpose of improving students’ communicative ability such as group work or pair work or other contextualized activities to help students learn grammar and vocabulary, we consider this as *focus-on-form* instruction in accordance with Ellis’ definition, as explained above. Some few occasions like recast without meaning-based activities are relegated into *focus-on-form*, because they take place when students are attempting to communicate. If teachers just explain grammar rules or the meaning of new words or phrases and then ask students to do some practice/exercises such as filling in the blanks, translating sentences from Chinese to English, or vice versa, by using these new grammar points, new words or phrases, and other practices or exercises in a decontextualized and structural way, we regard this type of instruction as *focus-on-formS* instruction. Meaning-based activities whose primary purpose is to practice specific linguistic forms rather than to communicate are categorized as *focus-on- formS* as well.

Since the establishment of a dichotomy of *focus-on-formS* and *focus-on-form* instruction according to Ellis’ conceptualization of form-focused instruction, there have been substantial studies in the field of second language acquisition (SLA) examining their effect and mixed results were obtained. Some studies demonstrated that *focus-on-form* instruction is more effective. For example, Shintani (2013) investigated the effect of two types of instruction on the acquisition of a set of nouns and adjectives by young Japanese children who were English beginners. The study reported that although both types of instruction were effective for the acquisition of nouns, *focus-on-form* instruction was more effective for the acquisition of adjectives. Burgess and Etherington (2002) examined teachers’ attitudes toward grammar teaching and learning within an English for academic purpose (EAP) context, where 48 EAP teachers in British university language centers were the participants. Results indicated that the majority of teachers in the study appreciated the value of grammar for their students and had a favorable attitude to the *focus-on-form* approach. In contrast, different research findings regarding the effectiveness of two approaches were also noted. Laufer (2006) compared the effectiveness of *focus-on-formS* and *focus-on-form* approaches to learning new L2 words by 158 high-school learners of English as an L2. She reported that both approaches were effective in learners’ acquisition of English vocabulary and *focus-on-formS* was claimed to be indispensable for L2 vocabulary learning. Norris and Ortega (2000) examined the effectiveness of L2 instruction by conducting a meta-analysis of experimental and quasi-experimental studies. One of the research findings was that the two approaches were equally effective in facilitating L2 learning. From what has been discussed, it is clear that mixed results about the effectiveness of *focus-on-formS* and *focus-on-form* instruction have been identified. Nonetheless, those studies were conducted in contexts, whose sociocultural milieus were very different. Little is known about how Chinese EFL teachers perceive these two methods in tertiary classrooms in terms of their effectiveness. One of the sporadic studies is Long and Liu (2007) explored Chinese university students’ cognitions about grammar teaching via a questionnaire. Their study revealed that their university English teachers tended to adopt *focus-on-formS* instruction when teaching grammar. It is noted that their study is based on a questionnaire and acquired self-reported cognitions from university students. However, how Chinese university English teachers approach grammar teaching in the classroom is unknown. Even less has been reported on how these EFL teachers conceptualize these challenges and what they really do in classroom teaching to promote EFL learning given the significance of English as a foreign language in the country. Such a study is especially important in the context of globalization, where many Chinese students are on study-abroad programmes through the medium of English in different parts of the world, where accurate and appropriate academic language use is paramount (Zhang, 2013). It is anticipated that findings from our study will shed light on how to meet the needs of not only domestic Chinese students but also Chinese students overseas through understanding of teachers’ cognitions about language teaching. We attempt to fill this research gap by addressing the following questions:

1. What are university English teachers’ cognitions about form-focused instruction in Chinese university classrooms?
2. How are university English teachers’ cognitions about form-focused instruction enacted in their actual classroom teaching?
3. How do we conceptualize Chinese university English teachers’ cognitions from a sociocultural theory?

## The Study

### Context

Participants in this study are chosen from a comprehensive university in central China, where arts, business, science, engineering, and medicine majors are offered. All students in this university have learned English for at least 9 years in their schooling education before being enrolled as university students. They are relatively proficient in English skills, belonging to advanced English learners in a general sense. In this university, English is taught as a compulsory course to non-English majors for the first 2 years, commonly known as College English. The compulsory all-embraced College English course is usually named *Comprehensive English*, where all language skills, including reading, writing, grammar, vocabulary, listening and speaking, are integrated, synthesized and condensed as one course. That is to say, university English teachers have to cover all of the above-mentioned language skills and knowledge in their classes. For each semester, there are 32 sessions and two sessions each week, totaling 64 h of in-class instruction. In a highly centralized education system, university English teachers in China normally have to teach according to the *Syllabus* enacted by Chinese MOE. The *Syllabus* stipulates that the goal of College English is to enable students to communicate in English accurately and fluently but gives priority to fluency and communication (Zhang, 2016). We have found that no matter what majors their students belong to, all teachers in this university used the same textbook and very few of them supplemented extra materials from which students could learn English. Regarding students’ assessment, students were evaluated by semester exams, and College English Test (CET) Band-4 or Band-6 administered by MOE, which were held throughout the whole country. A salient issue in English teaching is that the syllabus requirement enacted by MOE is at odds with the assessment methods, which still focus on testing students’ reading and rote learning skills.

### Participants

Four teachers volunteered to participate in this study after receiving the research invitation based on the principle of convenience sampling (Creswell, 2012). For the sake of anonymity and confidentiality, their pseudonyms, Wang, Zhang, Zheng and Liu, are used. Two are male while the other two are female. Three of them had a Master’s degree and the other one had a Bachelor’s degree. Among the four, two had more than 10 years’ teaching experience, while the other two had fewer than 10 years. It was also noticed that two teachers had overseas study experience, 12 months and 6 months, respectively, and the other two teachers did not have such experience. In terms of their professional qualification, three of them graduated from normal universities, which meant that they had some basic teacher education background; while only one graduated from a comprehensive university and did not go through any level of teacher training before embarking on teaching as a career. Their demographic information is presented in Table 1.

**TABLE 1:** Participants’ demographic information.

**Name**	**Gender**	**Age**	**Academic qualification**	**Teaching experience**	**Overseas study**	**Attended normal/teachers university (yes/no)**
Wang	Male	46	Master	10 years	12 months	Yes
Zhang	Female	33	Master	7 years	No	Yes
Zheng	Male	40	Bachelor	16 years	6 months	No
Liu	Female	28	Master	3 years	No	Yes

As is stated above, four college English teachers employed the same textbook named *Contemporary College English* (second edition) which was compiled by three Chinese scholars and published by Foreign Language Teaching and Research Press. There were 16 units in this book with each unit including *the text, notes on the text, glossary, preview, speaking, vocabulary, grammar*, and *writing*. For a typical unit, teachers took two sessions to complete it. Specifically, students had to do the *preview* part before going to in-class instruction at first. In the first session, teachers started with explaining new words in the *glossary* of each unit, then introduced the author and some background knowledge in the *notes* and finalized the *text* analysis by explaining the meaning of difficult sentences in the text, analyzing the text structure and its writing style. In the *glossary* part, teachers tended to explain the meaning of new words in both English and Chinese and then displayed several examples usually in the form of translating from English into Chinese to students where new words were embedded. In the second session, they went to the other parts of the text including *speaking, vocabulary*, and *grammar*, and asked students to do the writing after class. In the *vocabulary* part, teachers mainly asked students to do the exercise items which were usually designed in the form of filling in the blanks, translating from English into Chinese or from Chinese into English and then revealed so-called standard answers in teachers’ reference book to students. Students would raise questions if they still had difficulty in some items after they had obtained answers and teachers would explain problematic vocabulary items to them. In the *grammar* part, teachers started with examples, which were usually sentences in the text where some particular grammar points were used. They tended to present the grammar rules of that particular point and then asked students to do the exercises in a decontextualized way. Since all four teachers responded that they taught all units in this way, we only observed their two sessions of one unit teaching.

### Data Collection

Four university EFL teachers’ cognitions about form-focused instruction were explored and the research data were collected from them through three instruments as a way of triangulation: semi-structured interviews, classroom observations, and stimulated recall interviews over one academic semester.

#### Semi-Structured Interview

A semi-structured interview is a kind of interview directed by some general themes rather than specific questions, in which interviewees can freely talk about their ideas toward the pre-set themes by researchers (Given, 2008). A semi-structured interview is suitable for a study where a small number of respondents are interviewed deeply and where a researcher aims to capture some elements from natural conversation (Zhang, 2010; Creswell, 2012). Participants, in this study, were first interviewed once about their cognitions about form-focused instruction in their English teaching classroom, which lasted around 1 h for each interview (see Appendix 1). To avoid misunderstandings and acquire authentic and accurate messages, the language used for the interviews was Mandarin. All interviews were audio-recorded and conducted at places nominated by the participants, and then transcribed and translated verbatim by the first author. They were asked purposes of English language teaching in Chinese university classrooms, proper methods of implementing English language teaching in classes, and their preferred approaches to teaching English in their classes. We did not ask participants directly what cognitions they had about form-focused instruction but elicited their cognitions by analyzing and summarizing their responses to those questions. We did this in consideration of two points. One is we were afraid that participants did not understand the conceptualization of form-focused instruction and its subdivision *focus-on-formS* and *focus-on-form* and could not respond to our questions accurately. The other is that we did not want to raise questions to participants as a role of “experts,” which could easily embarrass participants who might feel ashamed for not knowing those terms. In order to create a natural conversation, we just had simple conversations with them about several topics surrounding English language teaching in a relaxed way so that they were willing to talk with us and give their genuine responses to our questions. By doing this, we intended to obtain reliable and valid data.

#### Class Observation

Observations are widely used in social science research as a data collection strategy (Creswell, 2012) and are extensively used in language teacher cognition research as well (Borg, 2015). Researchers, occasionally, need to understand teachers’ pedagogical actions, not only focusing on teachers’ thinking without taking their actions into consideration. Relying on classroom observations, researchers are able to collect direct information from their own observations rather than participants’ self-reported accounts (Dörnyei, 2007). Observational data provide research opportunities to gather information in a real context, enabling researchers to understand the context of a study, the information that participants’ unconsciously miss or which they cannot express in their words. In this study, after participants were interviewed, they were also asked to nominate one unit course (two sessions, 4 h) for us to observe. The first author attended their nominated classes and observed how participants implemented English teaching in their classrooms. The whole time of four participants’ classroom observation is 16 h. Owing to the requirement of university, the classes were not audio-recorded in case there were potential law or other problems; however, field notes were made and the whole class teaching was audio-recorded. In the field notes, participants’ teaching sections’ instruction time ranges especially vocabulary teaching and grammar teaching sections were noticeably marked so that we do not need to go through the entire lessons’ recordings in the immediate stimulated recall interviews. Additionally, documents including textbook and PowerPoint slides were also collected. The documents enabled us to understand teachers’ practices in vocabulary teaching and grammar teaching to a greater extent. Our purpose of classroom observation was to find out participants’ cognitions about grammar instruction and vocabulary teaching while they made particular decisions in their actual classroom teaching.

#### Stimulated Recall Interview

Classroom observations tend to be not the sole data collection strategy employed in language studies as they are insufficient as a means of gathering data in depth and verbal commentaries such as stimulated-recalls usually complement observations to guarantee the validity of the inferences (Duff, 2008). According to Calderhead (1981), stimulated recalls usually involve the use of videotapes or audiotapes of participants’ behavior, which are used later as a prompt to aid participants to reflect on their thought processes at the time of their behavior. Stimulated recalls generate verbal commentaries about cognitions occurring during previously performed behaviors by using a stimulus. Teachers cannot express their thinking concurrently with their teaching, hence, retrospective verbal accounts are required to explore their inner thoughts when they make interactive decision-making (Gass and Mackey, 2013). In this study, stimulated recall interviews referring to interviews where stimulated recalls are involved were employed to help participants recall their justification of their decision-making and their rationale underlying their classroom actions. Specifically, after a comparison was made between what they reported in the interviews and what was observed in classroom teaching and the congruence and incongruences between them were found, stimulated recall interviews were conducted with them to find out factors that contributed to “tensions” between their cognitions about form-focused instruction, as reported in the interviews and practices observed in their classroom instruction. Participants were stimulated recall interviewed once the first author finished their each nominated classroom teaching observation. Segments of their grammar instruction and vocabulary teaching were selected and the stimulated recall interview questions were prepared. Questions (see Appendix 2) like why you performed in a different way in classroom observations from what you reported in the interviews were raised to participants to find out the factors contributed to “tensions” between their cognitions about form-focused instruction reported in the interviews and reflected by their classroom instruction. Participants answered these questions with the help the tape recorder. The playback time of each interview is around 30 min and each interview lasted around 1.5 h. For four participants, we have around 240 min playtime and 12 h interviews in total.

### Data Analysis

The data for our study were qualitatively analyzed, mainly generated by a triangulation of methods including semi-structured interviews, classroom observations, and stimulated recall interviews, as mentioned above. The data analysis went through three stages according to the sequential data yielded from different ways of data collection and all stages’ data analysis almost underwent the same procedure. According to Creswell (2012), there are six steps in analyzing and interpreting qualitative data: (1) Collecting data, (2) preparing data for analysis, (3) reading through data, (4) coding data, (5) coding the test for description to be used in the research report, and (6) coding the test for themes to be used in the research report. We followed this principle in processing our data. To be specific, once data were collected successfully, a preparation for data analysis was made. First, all interviews and audio files (including those made in interviews, classroom observations and stimulated recall interviews) were fully transcribed and later, translated into English versions verbatim. All English versions were then returned to participants seeking their suggestions on whether the transcribed ideas genuinely reflected their ideas. All participants commented on the translation and provided their feedback on the English versions. After several rounds of revision of the English version, the final version was ready to be coded. Data from all the instruments were manually coded and analyzed, and the process was sequential and recursive. Both of two authors coded the data in order to improve coding reliability. If disputes on coding between us occurred, we recoded till we reached agreement. In the first two stages of data analysis, data generated from interviews and classroom observations were coded deductively by two terms of form-focused instruction, *focus-on-formS* or *focus-on-form*. As stated in the literature review part, if participants’ professed beliefs and their actual classroom instruction about vocabulary teaching and grammar teaching were undertaken in meaning-based activities like pair work, group work or other contextualized activities, we considered it as *focus-on-form* instruction; if participants only explained grammar rules or meanings of new words or phrases and then asked students to drill them in a way like filling in the blank and translation instead of asking them to practise new knowledge in meaning-based activities, we classified them as *focus-on-form*S instruction. Therefore, it is clear-cut to distinguish *focus-on-formS* from *focus-on-form* instruction with its judging principle lying in meaning-based activities or not. Following this, formal coding commenced with reading through the transcripts and a bottom-up open-coding approach was adopted (Ellis and Barkhuizen, 2005). Codes such as “pair work,” “group work,” “rules explanation,” “examples,” “filling in the blanks,” “translation from Chinese to English,” and “translation from English to Chinese” were used to categorize the emerging themes in teachers’ instruction procedures, which were further used to identify teachers’ teaching categories, *focus-on-formS* instruction or *focus-on-from* instruction. In the third stage, data yielded from stimulated recall interviews were coded. The first author carefully reviewed and coded the data to identify themes. Codes like “limited classroom time,” “the use of textbook,” “examination-orientation,” “students’ needs,” were used to yield the themes which guided us to seek teachers’ stated reasons for justifying the inconsistence between their reported *focus-on-form* instruction in the interviews and their actual *focus-on-formS* instruction reflected in classroom observations. Following the initial reading and coding, line-by-line coding was adopted in order to find more ideas for explaining the incongruence between their reported teaching methods and their actual teaching methods. As suggested by Charmaz (2006), line-by-line coding not only ensured themes grounded in the data, but helped avoid missing important themes. Finally, comprehensive and useful ideas were identified and sorted out in the files.

## Findings and Discussion

### Teachers’ Cognitions From Interviews

All four teachers expressed that they supported *focus-on-form* instruction in EFL teaching according to their responses. When they were asked whether they taught students’ grammar rules in class, they reported that grammar rules should be avoided and grammar points should be implemented in communicative or language-use contexts. They justified that university students had learned grammar systematically and explicitly in primary and secondary schools and there was no need to teach grammar to them deductively again. For example, in the following excerpt, Wang explained his perception about grammar instruction rather explicitly.

**Excerpt 1:**

I don’t think grammar rules should be presented in English classes. I believe university English teachers are supposed to teach students how to use grammar and language in the specific context in English teaching classes. I think it is more important to develop students’ communicative competence than emphasizing their grammatical accuracy solely (Interview, Wang).

The other teachers also had the same view as Wang. They responded that identifying purposes of grammar teaching was important for teachers before teaching grammar to students. Teachers should not teach grammar just because grammar *per se* is a core component of language. The significance of grammar teaching lies in teaching students how to use grammar in communicative activities, especially for advanced English learners such as university students. They went on explaining that students who were able to find out answers to the questions concerned with grammar items in discrete paper-based tests did not indicate that they were competent in, or had a good command of grammar knowledge. Instead, using grammar rules accurately and appropriately to communicate with others would demonstrate that they acquired solid grammar knowledge and skills. Therefore, students should be taught to learn grammatical points in specific communicative contexts. It can be inferred that their cognitions are conditioned by the way students make judgment on teachers. Results indicate that “becoming a good teacher depends on many cultural and linguistic assumptions that are linked to microaggression” (Maddamsetti et al., 2018, p. 148). This might be the reason why this particular teacher was so much focusing on student needs.

In the same vein, when they were asked about their ideal vocabulary teaching methods, they remarked that vocabulary should be taught in communicative texts or interactive dialogs. They did not like the traditional way in which teachers began with presenting new words in a decontextualized way, then interpreted the meaning of those words in Chinese and ended with asking students to use these words to do some translation practices. This method of vocabulary teaching is actually asking students to memorize vocabulary by repetition. They illuminated that rote learning of vocabulary was not the same effective as learning it in communicative context. In order to deepen students’ impression about English words and teach them how to use these words in their own writing and speaking, English vocabulary should be taught in communicative contexts. For example, Zhang stated her perception about form-focused instruction in vocabulary teaching in Excerpt 2.

**Excerpt 2:**

Personally, I think vocabulary teaching should be implemented in specific texts. Taught by this way, students are able to make sense of the meaning of words and know how to use them in their writing. Students only knowing English words’ spelling and their Chinese meaning are far from goals of learning English vocabulary. More importantly, they have to know how to represent them in their own outputs like speaking and writing (Interview, Zhang).

The other teachers expressed similar ideas with Zhang. They all stated that students should learn English words in meaningful contexts rather than in discrete sentences. They felt odd when seeing many university students memorize English words by holding bilingual vocabulary books which listed a series of words and their Chinese translation and several examples of using them.

To sum up, participants in the interviews were all in favor of *focus-on-form* instruction in both their grammar instruction and vocabulary teaching in Chinese university classrooms. The result is consistent with a number of previous studies stating that English teachers prefer *focus-on-form* instruction and contributes to a general agreement that the *focus-on-form* instruction is effective in grammar instruction and vocabulary teaching (Burgess and Etherington, 2002; Nassaji, 2013; Shintani, 2013; Saito and Wu, 2014; Spada et al., 2014; Afitska, 2015; Ellis, 2015).

### Teachers’ Cognitions From Classroom Observations

By observing teachers’ classroom teaching, we found that participants invariably adopted *focus-on-formS* instruction in teaching both grammar and vocabulary. This reflected that despite their reporting preferences for *focus-on-form* instruction as part of their belief system, teachers actually followed *focus-on-formS* while teaching grammar or vocabulary. Teachers sometimes did teach grammar explicitly to their students when they believed that their students had difficulty in understanding some particular grammar points or items. They tended to start with explaining grammar rules of those particular points to students and then gave several examples to show how grammatical points were used in some discrete sentences. The examples usually took the form of translating Chinese sentences into English, or vice versa, as shown below. Zheng explained the meaning of a difficult sentence in a text, by which he taught a grammar point.

**Episode 1:**

Teacher (T hereafter): There is an important language point here. Please look at the last sentence in paragraph 4:

Mattel refashioned the doll into a decent, all-American—although with an exaggerated breast size —version and named it after Barbara, who was then a teenager.

Do you notice this sentence has a special feature in language use?

Student (S hereafter): … (quiet, no response).

T: Please pay attention to *although with an exaggerated breast size*. This is a unique structure used in English grammar. That is, Conjunction + Preposition Phrase. This structure makes sentences concise and succinct. Next, I would like to give you an example to show how to use this grammatical point. For example,

Even though with the same educational background, he was better paid than his wife.

How to translate this sentence into Chinese?

S: … (in chorus).

T: Great! The same example is like, *even though with the same ability, men are better paid than women*.

In this episode, Zheng adopted a method similar to the traditional grammar-translation method in teaching grammar to students. First, he asked students to identify a grammar point used in the text in the textbook. He pointed out the grammar point by outlining its structural form after his students were unable to find it. He illustrated this point by asking students to translate a sentence where this language point was embedded. With an emphasis on its structure and the grammatical rule, his teaching method fostered students’ understanding about the grammar point. He was not observed to teach a grammar point in meaningful activities such as asking students to make a dialog to practice the grammar point. The remaining teachers also utilized the similar way of presenting grammar points to students in their classroom teaching. All of their grammar instruction did not involve meaningful activities but explained grammar rules and asked students to drill them.

Regarding vocabulary teaching in participants’ classroom teaching, they also employed *focus-on-formS* instruction. Specifically, teachers usually explained meanings of English words or phrases, followed by giving several examples of using these words and phrases to students. They did not put new words or phrases in real communicative contexts. For example, Liu presented her vocabulary teaching in this way.

**Episode 2:**

T: Today we are going to learn a new lesson. Please turn to Glossary page and we have to learn the new words before going to learn the text. The first word, *Associate, it is* an important word and it is a verb in this lesson. It means making a connection between people or things in your mind. We have to use active voice, not passive voice. Here is an example of showing the usage of *associate*

e.g., What do you associate with such a heavy snow? How to translate it into Chinese?

S:. (in chorus).

T: Nice. Please translate this sentence from Chinese into English by using *associate*.

…(a Chinese sentence).

S: ….We associate China with the Great Wall (in chorus).

T: Meanwhile, *associate* can also be an adjective and it means a lower rank in titles. Such as Associate Professor, Associate Dean, and Associate Editor …

Liu’s explanation of the meaning of a new word *associate* as a verb or an adjective was followed with several examples that used it. She asked students to translate Chinese sentences into English by using *associate*. This way is apparently a *focus-on-formS* approach. This reflects that the teacher’s approach to teaching vocabulary is actually *focus-on-formS* instruction. Their vocabulary teaching was conducted in a decontextualized way.

As is evident so far, teachers’ cognitions about form-focused instruction reflected in their practices in their actual grammar and vocabulary teaching is in fact *focus-on-formS* instruction. Different from their reported cognitions in their interviews, these teachers’ cognitions changed when they were teaching in the actual classroom. This is interesting. Such incongruence might demonstrate that teachers’ cognitions are not static but dynamic instead (Burns et al., 2015; Kubanyiova and Feryok, 2015). This suggests that teachers’ decision-making in the class, to a great extent, is reliant on the context where teaching activities take place. This is justified by SCT, which posits that teachers’ cognitions are quite likely to change due to the mediation of cultural, institutional, and historical contexts they live in. The available literature suggests that Chinese EFL teachers usually adopted an eclectic approach in their classroom teaching. This might have given them sufficient room to maneuver, making it possible for them to change their complex beliefs (Fang, 1996; Feryok, 2010; Li, 2013, 2017; Zheng, 2013; Xiang and Borg, 2014; Zhang and Liu, 2014; Zheng and Borg, 2014).

The findings from classroom observations as a whole suggest that teachers’ cognitions about teaching changed when the teaching environment changed because they had to make decisions in actual classrooms that were compounded by various factors such as students’ EFL proficiency, motivation, textbooks required to be used, the examination-oriented syllabus, limited class time, and many other factors. According to SCT, teachers’ cognition development is an interactive process, mediated by the social, cultural, and historical contexts. In this study, teachers’ cognitions about form-focused instruction are mainly mediated by institutional factors including their teaching environment and their students. Despite *focus-on-form* instruction was reported in their interviews as their preferred teaching approach, teachers tended to adopt *focus-on-formS* instruction in their classroom (see Ellis, 2012; Shegar et al., 2013).

### Factors Contributing to Changes in Teachers’ Cognitions

Since teachers’ cognitions changed when they conducted their classroom teaching, we scrutinized stimulated recall interviews for possible reasons contributing to the changes. When participants were asked why they changed their mind when they made decisions in their classroom instruction, they revealed that a plethora of sociocultural factors made them rethink how to teach grammar and vocabulary in the classroom. Such factors encompassed traditional Confucian thinking, limited instructional time, examination pressure, student needs, textbooks, and supervision team observation. These reasons are elaborated next.

First of all, all participants revealed that their conceptualization of education was heavily influenced by Confucian thinking. Impacted by Confucius theory and principle about teaching, education is viewed as a process of transmitting knowledge by teachers who are often considered as an authoritative figure by their students. In this teaching model, teachers usually dominate classroom teaching, interpreting and analyzing points of knowledge for students. Students are required or expected to mimic and regurgitate the knowledge delivered by their teachers to be evaluated by tests that are based on memorization of knowledge. Participants said they had been taught within this model and they themselves were quite comfortable with using the model to conduct their teaching, too. For example, Zheng said,

All Chinese teachers learned a well-known saying about a role of teachers in traditional Chinese education. In English, teachers have to teach students knowledge, tell students the way to live and answer students’ questions. Teachers have to be knowledgeable because they are a source of knowledge. This is just like a maxim saying that teachers have to have a full bucket of water to dispense if they want to give their students a bowl of water. It gives us an impression that teachers are knowledge-transmitters and are supposed to dominate in classroom teaching (Stimulated recall interview, Zheng).

Second, all of participants responded that the limited instructional time also restricted them from implementing teaching by following their cognitions that were reported in their interviews. They argued that they had too many language modules to complete in their classroom teaching, such as grammar, vocabulary, reading, translation, and writing. They did not have sufficient time to be assigned to grammar and vocabulary. They had to finish two modules fast so that they would have adequate time to work on other modules. A fast way for them was to explain grammar points and vocabulary explicitly and then give several examples to deepen students’ knowledge. To them, teaching grammar points and vocabulary in communicative contexts definitely takes more time than this direct instruction method. For example, Wang remarked,

I originally ask students to practise a grammar point in a communicative context. However, it is time consuming. Given we don’t have much classroom instruction time, I have to skip this step. You know we have to go through ten lessons with 12 weeks and 4 h for each week. The time is so precious for us and we have to use it meticulously (Stimulated recall interview, Wang).

Third, of all the four teachers, except Liu, three of them stated that compared to *focus-on-form* instruction, *focus-on-formS* instruction was more effective for improving students’ marks in discrete-point exams such as the nation-wide College English Test (CET) Band 4 and CET Band 6. As all Chinese university students had to sit CET 4 during their university study, teachers usually had a task to prepare students for them to successfully pass the test. Understandably, they adopted *focus-on-formS* instruction due to its advantage in helping students achieve good results. For example, Zhang said,

Undoubtedly, *focus on formS* grammar teaching instruction is more effective for exam-oriented students than *focus on form*. The accuracy of language is emphasized by *focus on formS* instruction, while the fluency of language is underscored by *focus on form* instruction. When CET 4 and CET 6 were first introduced to university students, it was an obligation that all undergraduates had to pass CET 4 before they were awarded by their bachelor degree certificates. If they failed, they were not accorded with bachelor degree certificates. Therefore, many students study English with a sole purpose of passing CET 4. In order to prepare students for CET 4, I have to use *focus on formS* instruction (Stimulated recall Interview, Zhang).

Two other teachers, Zheng and Liu, responded that they adopted *focus-on-formS* instruction on account of students’ needs. They said they gave primacy to students’ needs in implementing their classroom teaching. Though they attached importance to developing students’ communicative competence, students did not always agree with them. Many students told them that they wanted to learn more knowledge from them and they felt to have learned nothing from pair work or group work. For example, Liu said,

I know learning English for communication is important for students. Communication should be the final purpose of learning a language for students. However, some students don’t hold the same opinion with me. They told me that they wanted to learn more knowledge from the class. If teachers only ask students to do some pair work or group work or other classroom activities, they find that they achieve nothing from classroom teaching (Stimulated recall interview, Liu).

Another two teachers, Wang and Zhang, ascribed their cognitions’ changes to the textbooks they had to use in classroom teaching. They responded that despite the fact that they had their own teaching principles, they were not able to implement them in classroom teaching at will. In China, English language teaching is often textbook-based and teachers teach English to students by closely following the prescribed textbooks. Most books are structure-based, which makes it difficult for teachers to use them in communicative ways. For example, Wang remarked,

The textbook I used do not encourage me to teach grammar and vocabulary in communicative contexts. My dean demands us to cover all ten lessons in our textbook because some contents in the textbook will be included in semester test. If I use my own way to teach English, the result is I cannot complete all lessons in the textbook. My students will suffer from this, because I do not deliver all textbook contents to them. They will feel unfair and depressed (Stimulated recall interview, Wang).

Last but not least, one participant, Teacher Liu, revealed that she used *focus-on-formS* instruction to teach grammar points due to the presence of a supervision team member in her class. As she said in the interview, all young teachers in her university had to be observed by one or two supervision team members who were usually senior and retired teachers. After observing, they graded these teachers in the evaluation forms provided and pointed out their shortcomings. In order not to be evaluated by them negatively, she and her fellow colleagues always did their utmost to satisfy those senior teachers, whose teaching methodology was usually old-fashioned, with a clear focus on *focus-on-formS* or grammar. Because of their anxiety related to possible negative evaluations by these veteran teachers, young English teachers had to adhere to the familiar procedure that is typical in the sequence of “Presentation-Practice-Production.” Teacher Liu wanted to be evaluated positively, so she adopted a method that those veteran teachers were familiar with and would prefer.

As shown in the analysis above, a multitude of sociocultural factors contributing to the changes in teachers’ cognitions in their actual classroom teaching were mentioned by the participating teachers. According to SCT, teachers’ cognitions are quite likely to change due to a consideration of multiple sociocultural factors. In this study, these factors encompass traditional Confucian thinking, limited instructional time, examination pressure, students’ needs, textbooks, and supervision team observation. Such findings are not unfamiliar. A large literature base can be easily resorted to, where researchers have reported that teachers’ cognitions are easily subject to the influence of contextual factors (Fang, 1996; Farrell and Bennis, 2013; Borg, 2015; Farrell and Ives, 2015; Jiang et al., 2020). Therefore, in this sense, our study lends further evidence to the findings reported in other similar contexts (Lee, 2009; Mak, 2011; Bao et al., 2016; Li, 2017; Gao and Zhang, 2020).

## Conclusion

The purpose of this study was to understand four Chinese university EFL teachers’ cognitions about *focus-on-form* instruction in English teaching classrooms from a sociocultural approach. The research yielded a substantial amount of information that can provide insights into effective language teaching. Firstly, four teachers reported that they were in favor of *focus-on-form* instruction, especially in grammar and vocabulary teaching. They believed that English at the tertiary level should be taught in meaningful communicative contexts. However, our classroom observations show that these teachers changed their classroom decisions due to a large number of sociocultural factors, especially their deep-rooted Confucian thinking on teaching, by which knowledgeable teachers are competent teachers. Our data, despite limited, point to a conclusion that these Chinese EFL university teachers’ cognitions are complex and dynamic, greatly shaped by contextual factors. Teachers’ classroom decision-making is not a simple matter of cognition-determined activity. It is influenced by many cultural, institutional and historical factors that are related to the whole enterprise of teaching and learning.

Our findings have several practical implications for Chinese EFL teachers and stakeholders such as government policy-makers. Chinese EFL teachers should be aware that their cognitions can be easily changed by a plethora of contextual factors. Therefore, they need to plan their teaching, with possible sociocultural factors having to be taken into consideration. They have to be reminded that it is likely that teaching itself is a complex undertaking and that because of this understanding they are not able to put their original cognitions into practice in their actual teaching. They also need to constantly improve themselves so that they are up to date to the latest knowledge and skills that will enable them to formulate more realistic cognitions about EFL teaching, with a good understanding of the importance of context-informed practice owing to educational practices with Chinese characteristics and a distinctive socio-educational environment (Sun, 2017; Gao et al., 2020; Li et al., 2020). For instance, they should proactively take part in teacher professional development seminars or workshops, where they will be afforded the opportunity to personally witness how potentially innovative teaching methods are used by colleagues through concrete examples so that they are able to understand them in a holistic way and apply them in their own everyday pedagogical practice. The findings from our study clearly suggest that Chinese university EFL teachers need to attend workshops or professional learning courses. Through these learning opportunities, experienced teachers can share with them how a scaffolded approach to form-focused instruction can be effectively delivered and communicative competence is not separated from focus-on-formS, and how these teachers in re-training or professional development can embrace grammar teaching in the context of meaning-focused communication (Zhang and Zhang, 2020).

Additionally, Chinese universities should create a favorable environment and provide full support to teachers. Limited classroom time, textbook-orientated English teaching and examination-oriented evaluation system were indicated by participants as factors that confined them and restricted their implementation of their original cognitions about effective grammar instruction. Universities should give autonomy to teachers for choosing teaching materials so that teachers can make plans accordingly and make full use of the limited instruction time. The evaluation system might also need to be changed in order for students to learn language skills that they can put into use. Relying on discrete-point tests as the only evaluation method is counter-productive. Having said all this, we need to stress that our findings need to be understood with caution. Our findings are based on case studies, which means that we reported on only four teachers’ cognitions about form-focused instruction in English language teaching. Therefore, the generalization of the study is severely restricted. Future research might need to consider using a mixed-methods approach in order to get general understanding as well as in-depth knowledge of Chinese EFL teachers’ cognitions about form-focused instruction in the provision of English language education.

## Data Availability Statement

The original contributions presented in the study are included in the article/supplementary material, further inquiries can be directed to the corresponding author/s.

## Ethics Statement

The studies involving human participants were reviewed and approved by the University of Auckland Human Participants Ethics Committee, with QS as the first author. The patients/participants provided their written informed consent to participate in this study.

## Author Contributions

QS and LZ conceived and designed the study. QS collected the data and drafted the manuscript. Both the authors revised the manuscript and got it ready for submission.

## Conflict of Interest

The authors declare that the research was conducted in the absence of any commercial or financial relationships that could be construed as a potential conflict of interest.

## Appendices

### Appendix 1 | Semi-Structured Interview Questions

List of basic questions (additional questions may be asked in relation to the teachers’ answers to the basic questions).

• Would you like to introduce your teaching and your university briefly?
• Could you please describe your own experiences of English language learning?
• Could you describe how you teach in your English classes? What is your purpose of English language teaching in classrooms?
• What do you think about the role of grammar in English language teaching?
• How do you teach grammar in the class? Do you teach grammar rules in class? Do you think that grammar should be taught in meaningful activities like pair work or group work?
• Do you teach vocabulary in your English classes and please describe how you teach vocabulary in your classes. Do you think that vocabulary should be taught in meaningful activities like pair work or group work?
• Tell me one of your typical English language lessons (how do you teach and why do you teach in that way). Please tell me your ideal English language teaching, especially how to teach vocabulary and grammar.

### Appendix 2 | A Part of Stimulated Recall Interview Questions

Let us talk something about your grammar instruction and vocabulary teaching with the help of the tape recorder.

• In your interview you said that grammar rules should be avoided, but why you did explain grammar rules to students in classroom?
• Could you explain why you did not design meaningful activities to practice grammar points but asked students to drill them?
• In your interview, you stated that new words should be taught in a meaningful context, why you only taught vocabulary in the Glossary not in specific context?
• Why did you always ask students to translate sentences by using new words instead of asking them to work on a meaningful activity with new words?
• Please explain why there is a gap between your ideal grammar instruction and vocabulary teaching, and your actual decisions in classrooms.
